# Trends in Hyperkalemia‐Related Sudden Cardiac Death Among Older Adults in the United States, 1999–2020

**DOI:** 10.1002/clc.70483

**Published:** 2026-10-06

**Authors:** Ibrahim Nagmeldin Hassan, Mohamed Ibrahim, Siddig Yaqub, Muhsin Ibrahim, Nagmeldin Abuassa

**Affiliations:** ^1^ University of Khartoum Faculty of Medicine Khartoum Sudan; ^2^ Al‐Neelain University Faculty of Medicine Khartoum Sudan; ^3^ Faculty of Medicine Karary University Khartoum Sudan; ^4^ Faculty of Medicine Sudan University of Science and Technology Khartoum Sudan

**Keywords:** CDC WONDER, epidemiology, hyperkalemia, mortality trends, older adults, sudden cardiac death

## Abstract

**Background:**

Hyperkalemia is a potentially life‐threatening electrolyte disturbance, particularly in older adults with comorbidities. Hyperkalemia can trigger fatal arrhythmias and contribute to sudden cardiac death (SCD). However, national trends in hyperkalemia‐related SCD remain poorly defined, especially in the aging US population.

**Methods:**

We conducted a cross‐sectional analysis using CDC WONDER mortality data from 1999 to 2020. Deaths were identified using ICD‐10 codes for SCD (I46.1, I46.9) as the underlying cause and hyperkalemia (E87.5) as a contributing cause. Age‐adjusted mortality rates (AAMRs) were calculated using the 2000 US standard population. Joinpoint regression was used to assess temporal trends and estimate annual percentage changes (APCs). Analyses were stratified by sex, age, race/ethnicity, region, urbanization, and state.

**Results:**

Between 1999 and 2020, 36 607 hyperkalemia‐related SCD deaths were recorded. The national AAMR more than doubled, from 0.65 to 1.32 per 100 000. Mortality was stable from 1999 to 2009 (APC –1.09%, *p* = 0.0566) but rose significantly from 2010 to 2020 (APC +5.02%, *p* < 0.0001). Males, Black individuals, and residents of the South and Midwest had the highest AAMRs and steepest increases. Rural areas consistently showed higher mortality than urban ones. Though most deaths occurred in older adults, significant increases were observed in younger adults (25–44 years) in the last decade. Nevada, Rhode Island, and California had the highest state‐level rates.

**Conclusion:**

Hyperkalemia‐related SCD mortality has risen substantially, with marked disparities by sex, race, geography, and age. These trends underscore the need for improved potassium monitoring, risk stratification, and equitable healthcare access to reduce preventable deaths.

## Graphical abstract

Hyperkalemia‐related sudden cardiac death (SCD) rates in US adults more than doubled from 1999 to 2020, with sharp increases after 2010. The burden was greatest among males, Black individuals, rural residents, and, surprisingly, younger adults—highlighting emerging disparities and the urgent need for improved potassium monitoring and chronic disease management.

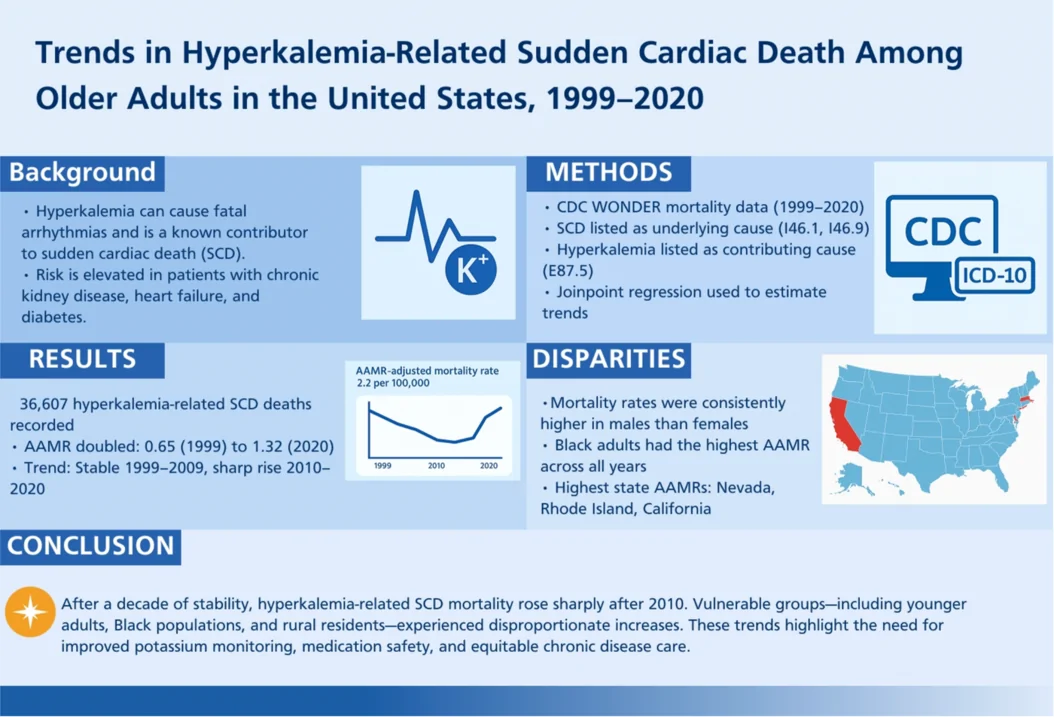

**Abbreviations**
AAMR: age‐adjusted mortality rate
AAPC: average annual percentage change
ACE: angiotensin‐converting enzyme
APC: annual percentage change
ARB: angiotensin receptor blocker
CDC: Centers for Disease Control and Prevention
CI: confidence interval
CKD: chronic kidney disease
CMS: Centers for Medicare & Medicaid Services
CR: crude mortality rate
DM: diabetes mellitus
ECG: electrocardiogram
HF: heart failure
ICD‐10: International Classification of Diseases, 10th Revision
MRA: mineralocorticoid receptor antagonist
NCHS: National Center for Health Statistics
RAAS: renin‐angiotensin‐aldosterone system
RAASi: renin‐angiotensin‐aldosterone system inhibitor
SCD: sudden cardiac death
STROBE: Strengthening the Reporting of Observational Studies in Epidemiology
U.S.: United States
WONDER: Wide‐ranging Online Data for Epidemiologic Research

## 1 Introduction

Hyperkalemia, defined as a serum potassium concentration exceeding 5.0 mmol/L, is a potentially life‐threatening electrolyte disorder that disproportionately affects older adults due to age‐related decline in renal function and a high burden of comorbidities such as chronic kidney disease (CKD), heart failure (HF), and diabetes mellitus (DM) [1, 2]. The kidneys play a central role in potassium homeostasis, and diminished glomerular filtration rate with advancing age impairs potassium excretion, thus predisposing older individuals to hyperkalemia [1].

In addition to physiological changes, polypharmacy is common in older populations. Medications that interfere with potassium excretion—particularly renin‐angiotensin‐aldosterone system inhibitors (RAASi), mineralocorticoid receptor antagonists, and certain β‐blockers—are frequently prescribed in the setting of CKD or HF, and are well‐established contributors to hyperkalemia [3]. This disturbance is not only a biochemical abnormality but also a significant clinical concern, as elevated serum potassium levels can provoke fatal cardiac arrhythmias and are a known precipitant of sudden cardiac death (SCD) [4].

Several epidemiologic studies have highlighted the association between hyperkalemia and increased risk of cardiovascular mortality, particularly in patients with renal dysfunction [5]. One study demonstrated that patients with CKD and serum potassium levels above 5.0 mmol/L had a significantly higher risk of SCD, suggesting a synergistic interplay between hyperkalemia and underlying organ dysfunction [5]. Moreover, even transient episodes of hyperkalemia during hospitalization have been linked to increased in‐hospital and long‐term mortality [6].

Despite the clinical importance of this condition, national‐level trend data on hyperkalemia‐related SCD—especially in the aging US population—remain lacking. This is particularly concerning given demographic shifts toward an older population and increasing prevalence of CKD and HF. Timely surveillance of these mortality trends is essential to inform clinical practice, improve patient safety, and guide policy interventions [7, 8]. Therefore, this study aims to evaluate long‐term trends in hyperkalemia‐related SCD among adults in the United States from 1999 to 2020, using nationally representative mortality data.

## 2 Methods

### 2.1 Study Design

We conducted a cross‐sectional analysis using mortality data from the Centers for Disease Control and Prevention (CDC) Wide‐ranging Online Data for Epidemiologic Research (WONDER) platform [9]. This publicly available database compiles death certificate data from across the United States. We analyzed data from 1999 to 2020 to evaluate trends in mortality related to hyperkalemia‐associated SCD among adults aged 25 years and older.

Deaths were identified using International Classification of Diseases, Tenth Revision (ICD‐10) codes [10]. SCD was identified when I46.1 (SCD, so described) or I46.9 (cardiac arrest, unspecified) was listed as the underlying cause of death, and hyperkalemia (E87.5–disorder of potassium metabolism) was simultaneously recorded as a contributing cause. Only records meeting both criteria were included in the final data set (Supporting Information S1: Table S1).

Institutional Review Board approval was not required due to the use of de‐identified, publicly accessible data. This study follows the Strengthening the Reporting of Observational Studies in Epidemiology (STROBE) guidelines for cross‐sectional studies [11] (Supporting Information S1: Table S2).

### 2.2 Data Extraction

Data were extracted for the overall US adult population and stratified by demographic and geographic characteristics, including sex, age (in 10‐year intervals from 25–34 to ≥ 85), race/ethnicity, US Census Region (Northeast, Midwest, South, West), and urbanization category.

Race and ethnicity on death certificates were reported by funeral directors based on information provided by the decedent's next of kin. Urban‐rural classification followed the 2013 National Center for Health Statistics (NCHS) Urban–Rural Classification Scheme for Counties, where metropolitan areas (≥ 50 000 population) were categorized as urban and nonmetropolitan areas (< 50 000 population) as rural [12].

In accordance with CDC WONDER reporting standards, data points labeled as “unreliable” due to small cell counts or unstable estimates were excluded from analysis. Additionally, any observations with missing values for age‐adjusted or crude mortality rates (CR), or incomplete demographic classification, were omitted. These exclusions were minimal and did not materially impact the completeness or interpretability of subgroup analyses.

### 2.3 Statistical Analysis

Crude death counts and population estimates for each year were obtained from CDC WONDER. Age‐adjusted mortality rates (AAMRs) were calculated using the direct method, standardized to the 2000 US standard population. AAMRs were reported per 100 000 population, along with corresponding 95% confidence intervals (CIs) based on Poisson distributions.

To examine temporal trends, we applied log‐linear regression models to calculate annual percentage change (APC) for each subgroup and time segment (1999–2009 and 2010–2020). Where appropriate, we also computed average annual percentage change (AAPC) across the entire study period as a weighted mean of the APCs. A Monte−Carlo permutation test was used to identify significant joinpoints (inflection points) in trend segments. Statistical significance was defined as *p* < 0.05.

Joinpoint regression modeling was performed using version 5.0.2 of the Joinpoint Regression Program developed by the Statistical Research and Applications Branch of the National Cancer Institute [13]. This method identifies inflection points—referred to as joinpoints—where statistically significant changes in mortality trends occur over time. The analysis begins with the simplest model (no joinpoints) and sequentially tests whether adding joinpoints improves model fit, using a series of Monte Carlo permutation tests [14]. For each segment identified, the APC in the AAMR was estimated along with corresponding 95% CIs.

For comparative analysis, we evaluated differences in AAMRs and APCs between demographic subgroups (e.g., sex, region, urbanization). Parallelism testing of regression slopes was conducted to assess whether time trends differed significantly between groups. All data analyses and visualizations were performed using Python (v3.10) with statsmodels and seaborn libraries. To assess potential artifacts related to coding changes, we reviewed contemporaneous CDC WONDER metadata and found no known ICD coding transitions or systemic revisions in hyperkalemia or SCD classification between 1999 and 2020.

## 3 Results

### 3.1 Overall

Between 1999 and 2020, a total of 36 607 deaths from hyperkalemia‐related SCD were recorded among US adults. This cumulative toll reflects the growing clinical burden of hyperkalemia as a contributing factor in cardiac mortality.

From 1999 to 2020, the AAMR for hyperkalemia‐related SCD in the United States exhibited a dynamic pattern. In 1999, the national AAMR was 0.65 per 100 000 population (95% CI: 0.63–0.67). This rate remained relatively stable during the early 2000s, fluctuating between 0.61 and 0.68 until 2009. However, from 2010 onward, a sharp increase was observed, with the AAMR reaching 1.32 per 100 000 in 2020 (95% CI: 1.29–1.35). This represents a doubling of the mortality burden over the study period and highlights a shifting epidemiologic profile for hyperkalemia‐related cardiac deaths (Table 1; Figure 1).

**Table 1:** Demographic characteristics of deaths due to hyperkalemia‐related sudden cardiac death among older adults in the United States from 1999 to 2020.

Characteristics	Deaths (%)	AAMR (95% CI) per 100 000
Entire cohort	36 607 (100%)	0.79 (0.78−0.80)
Gender		
Female	17 389 (47.5)	0.66 (0.65−0.67)
Male	19 218 (52.5%)	0.95 (0.93−0.96)
Census region		
Northeast	7732 (21.1%)	0.82 (0.81−0.84)
Midwest	5862 (16.0%)	0.56 (0.54−0.57)
South	12 439 (33.9%)	0.72 (0.71−0.73)
West	10 574 (28.8%)	1.03 (1.01−1.05)
Race/ethnicity		
NH American Indian or Alaska Native	499 (1.3%)	1.32 (1.20−1.44)
NH Asian or Pacific Islander	1595 (4.3%)	0.86 (0.82−0.90)
NH Black or African American	6915 (18.8%)	1.44 (1.41−1.48)
NH White	27 598 (75.3%)	0.68 (0.67−0.68)
Hispanic or Latino	3865 (10.5%)	1.01 (0.98−1.05)
Urbanization		
Metropolitan (Urban)	29 705 (81.1%)	0.77 (0.76−0.78)
Non‐metropolitan (Rural)	6902 (18.8%)	0.83 (0.81−0.85)
Ten‐year age groups (years)^a^		
25−34	659 (1.8%)	0.07 (0.07−0.08
35−44	1498 (4.0%)	0.16 (0.15−0.17)
45−54	3496 (9.5%)	0.38 (0.36−0.39)
55−64	6567 (17.9%)	0.86 (0.84−0.88)
65−74	8417 (22.9%)	1.65 (1.61−1.68)
75−84	8905 (24.3%)	2.98 (2.92−3.05)
85+	7065 (19.3%)	5.91 (5.77−6.05)

Abbreviations: AAMR = age‐adjusted mortality rate, NH = non‐Hispanic.

a Crude mortality rate (CR) is used for analysis instead of age‐adjusted mortality rates (AAMR) for age groups.

**Figure 1:**
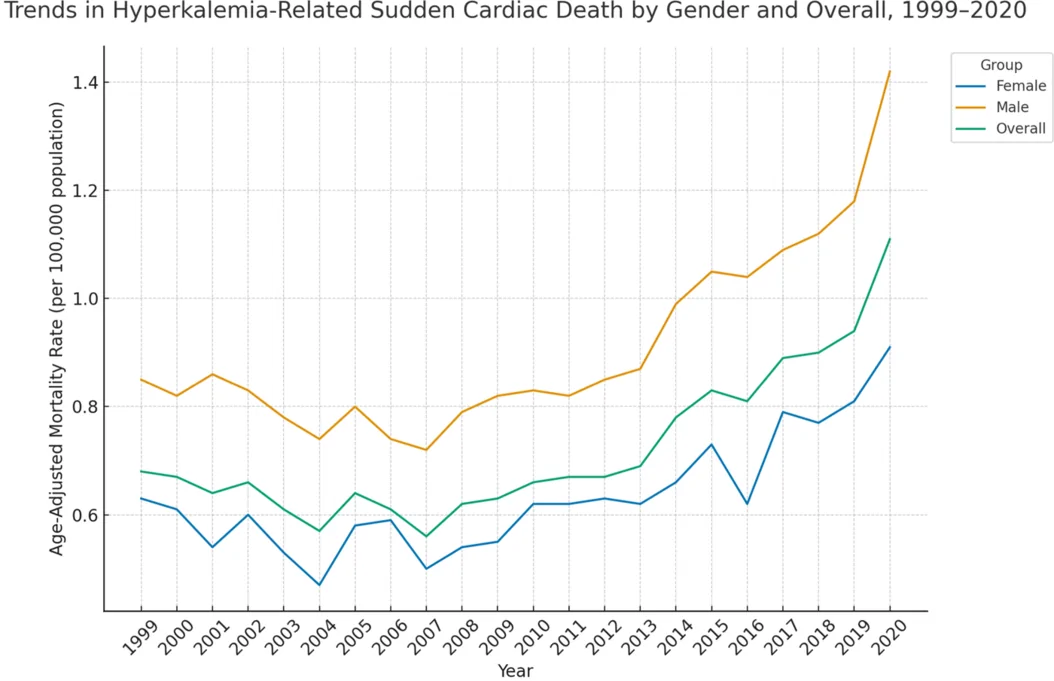
Trends in age‐adjusted mortality rates for hyperkalemia‐related sudden cardiac death stratified by gender and overall among older adults, 1999–2020.

Segmented analysis of the AAPC confirmed this shift. From 1999 to 2009, the APC showed a mild, borderline significant decline of –1.09% per year (95% CI: –2.05 to –0.11, *p* = 0.0566), suggesting stable or slightly improving outcomes in the early period. In contrast, the decade from 2010 to 2020 exhibited a significant and sustained increase in mortality, with an APC of +5.02% per year (95% CI: +4.13 to +5.91, *p* < 0.0001) (Table 2; Figure 1).

**Table 2:** Annual percentage changes (APCs) and average annual percentage changes (AAPCs) in mortality rate due to hyperkalemia‐related sudden cardiac death among older adults in the United States from 1999 to 2020.

Characteristics	Trend segment	Year interval	APC (95% CI)	AAPC (95% CI)	*p* value
Entire cohort				+1.33% (0.60% to 2.07%)	
	1	1999–2007	−2.05% (−3.14 to −0.95)		0.0083
	2	2008–2013	+2.18% (1.59 to 2.77)		0.0019
	3	2014–2020	+5.09% (3.32 to 6.89)		0.0023
Gender					
Female				+3.22% (1.40% to 5.08%)	
	1	1999–2003	−3.56% (−7.05 to 0.07)		0.1501
	2	2004–2006	+12.04% (0.44 to 24.98)		0.2903
	3	2007–2020	+3.92% (3.10 to 4.74)		< 0.0001
Male				+1.20% (0.42% to 1.98%)	
	1	1999–2007	−2.03% (−2.89 to −1.16)		0.0026
	2	2008–2013	+1.67% (1.02 to 2.32)		0.0071
	3	2014–2020	+5.09% (2.93 to 7.29)		0.0054
Census region					
Northeast				+3.70% (2.88% to 4.52%)	
	1	1999–2006	−0.83% (−2.39 to 0.75)		0.3396
	2	2007–2010	+10.14% (7.91 to 12.42)		0.0115
	3	2011–2020	+4.91% (4.00 to 5.84)		< 0.0001
Midwest				+1.35% (0.36% to 2.36%)	
	1	1999–2004	−3.13% (−5.46 to −0.75)		0.0624
	2	2005–2008	−7.76% (−8.89 to −6.61)		0.0061
	3	2009–2020	+6.98% (5.63 to 8.35)		< 0.0001
South				+3.04% (2.17% to 3.92%)	
	1	1999–2003	−4.29% (−6.46 to −2.07)		0.0330
	2	2004–2006	+10.26% (6.46 to 14.19)		0.1153
	3	2007–2020	+4.27% (3.51 to 5.04)		< 0.0001
West				+0.96% (0.30% to 1.61%)	
	1	1999–2012	−1.68% (−2.42 to −0.93)		0.0009
	2	2013–2020	+5.74% (4.48 to 7.02)		0.0001
Race/ethnicity					
NH American Indian or Alaska Native				+3.24% (0.21% to 6.36%)	
	1	2004–2012	−1.28% (−3.97 to 1.48)		0.4271
	2	2013–2015	+25.53% (19.29 to 32.09)		0.0725
	3	2016–2020	−0.49% (−8.40 to 8.09)		0.9142
NH Asian or Pacific Islander				−3.52% (−5.56% to −1.44%)	
	1	1999–2006	−6.52% (−10.28 to −2.61)		0.0181
	2	2007–2016	−3.27% (−5.47 to −1.02)		0.0222
	3	2017–2020	+2.10% (−3.93 to 8.50)		0.5725
NH Black or African American				+4.84% (2.95% to 6.77%)	
	1	1999–2006	−0.53% (−2.55 to 1.53)		0.6272
	2	2007–2017	+4.41% (3.32 to 5.51)		< 0.0001
	3	2018–2020	+22.47% (9.15 to 37.42)		0.1796
NH White				+3.10% (2.21% to 4.00%)	
	1	1999–2006			0.1293
	2	2007–2010			0.0263
	3	2011–2020			< 0.0001
Hispanic or Latino				+0.61% (−0.81% to 2.05%)	
	1	1999–2012	−2.07% (−3.04 to −1.09)		0.0014
	2	2013–2020	+5.48% (1.86 to 9.23)		0.0242
Urbanization					
Metropolitan (urban)				+3.21% (2.56% to 3.87%)	
	1	1999–2006	−1.12% (−2.38 to 0.16)		0.1380
	2	2007–2017	+4.01% (3.32 to 4.71)		< 0.0001
	3	2018–2020	+12.51% (10.27 to 14.81)		0.0554
Non‐metropolitan (rural)				+3.22% (2.08% to 4.38%)	
	1	1999–2006	+0.22% (−2.09 to 2.58)		0.8598
	2	2007–2020	+4.98% (3.80 to 6.17)		< 0.0001
Ten‐year age groups^a^					
25−34 years				−0.21% (−3.18% to 2.84%)	
	1	1999–2007	−2.49% (−4.72 to −0.20)		0.0866
	2	2009–2016	−0.86% (−4.64 to 3.06)		0.6766
	3	2017–2020	+6.48% (−6.25 to 20.94)		0.4355
35−44 years				+2.04% (0.48% to 3.63%)	
	1	1999–2011	−1.75% (−3.37 to −0.10)		0.0623
	2	2012–2020	+7.78% (4.70 to 10.95)		0.0015
45−54 years				+3.95% (2.48% to 5.44%)	
	1	1999–2014	+2.35% (1.44 to 3.26)		0.0002
	2	2015–2020	+8.33% (3.41 to 13.50)		0.0281
55−64 years				+4.56% (3.58% to 5.55%)	
	1	1999–2006	+1.83% (−0.31 to 4.00)		0.1449
	2	2007–2020	+6.15% (5.24 to 7.06)		< 0.0001
65−74 years				+1.37% (0.51% to 2.24%)	
	1	1999–2007	−3.55% (−5.08 to −2.00)		0.0030
	2	2008–2020	+4.93% (3.96 to 5.92)		< 0.0001
75−84 years				+1.60% (0.76% to 2.44%)	
	1	1999–2007	−2.75% (−4.16 to −1.31)		0.0075
	2	2008–2015	+3.37% (1.86 to 4.89)		0.0044
	3	2016–2020	+6.91% (6.05 to 7.78)		0.0005
85+ years					
	1	1999–2004	−4.29% (−5.62 to −2.94)		0.0036
	2	2005–2010	+1.26% (−1.60 to 4.21)		0.4397
	3	2011–2020	+2.17% (1.09 to 3.26)		0.0043

Abbreviations: AAMR = age‐adjusted mortality rate, AAPC = average annual percentage change, APC = annual percentage change, NH = non‐Hispanic.

a Crude rate is used for all age‐dependent analysis.

### 3.2 Trends by Gender

Between 1999 and 2020, the AAMRs for hyperkalemia‐related SCD were consistently higher in males compared to females. Among females, AAMRs ranged from 0.61 (95% CI: 0.56–0.65) per 100 000 in 2001 to 0.96 (95% CI: 0.91–1.02) in 2020, showing a modest rise over time. In contrast, males exhibited a steeper increase, with AAMRs rising from 0.86 (95% CI: 0.80–0.92) in 2001 to 1.67 (95% CI: 1.60–1.75) per 100 000 in 2020, highlighting a growing gender gap in mortality burden (Figure 1).

Segmented trend analysis revealed two distinct temporal phases. From 1999 to 2009, females experienced a nonsignificant decline in AAMRs with an AAPC of –1.21% (95% CI: –2.93% to +0.51%, *p* = 0.1596), while males showed a similar nonsignificant decrease at an APC of –0.96% (95% CI: –2.05% to +0.14%, *p* = 0.0839). However, between 2010 and 2020, both groups exhibited statistically significant increases. Females had an APC of +3.71% (95% CI: +1.91% to +5.55%, *p* = 0.0004), while males experienced a sharper rise with an APC of +5.10% (95% CI: +3.59% to +6.62%, *p* < 0.0001) (Table 2; Figure 1).

### 3.3 Trends by Race/Ethnicity

Between 1999 and 2020, hyperkalemia‐related SCD exhibited substantial disparities across racial and ethnic groups in the United States. Among these groups, Black or African American individuals consistently had the highest AAMRs, rising from 1.24 per 100 000 in 1999 (95% CI: 1.17–1.32) to 2.30 per 100 000 in 2020 (95% CI: 2.21–2.39). American Indian or Alaska Native individuals followed, with AAMRs increasing from 0.91 (95% CI: 0.75–1.10) to 1.40 (95% CI: 1.22–1.60) over the same period. White individuals demonstrated a consistent upward trend as well, with AAMRs nearly doubling from 0.69 (95% CI: 0.67–0.71) in 1999 to 1.31 (95% CI: 1.28–1.34) in 2020. Hispanic or Latino individuals experienced a similar rise, with AAMRs climbing from 0.66 (95% CI: 0.60–0.73) to 1.19 (95% CI: 1.12–1.26). Throughout the study period, Asian or Pacific Islander individuals maintained the lowest mortality rates, though these also rose from 0.55 (95% CI: 0.49–0.61) to 0.89 (95% CI: 0.82–0.97) (Figure 2).

**Figure 2:**
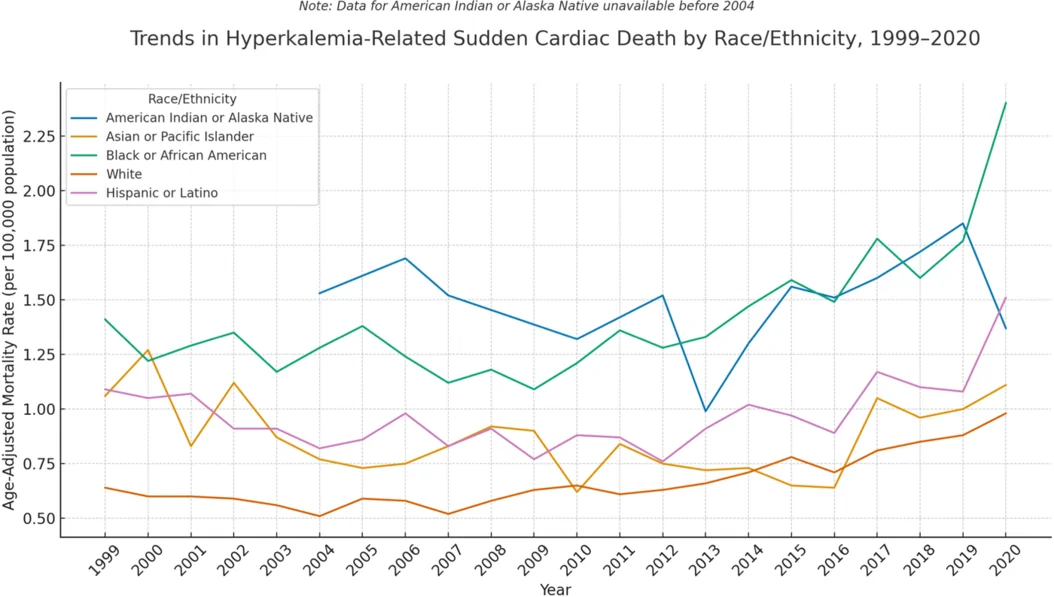
Trends in age‐adjusted mortality rates for hyperkalemia‐related sudden cardiac death stratified by race and ethnicity among older adults, 1999–2020 (*Note:* Data for American Indian or Alaska Native unavailable before 2004).

Trends in the AAPC revealed a marked shift between the two decades. From 1999 to 2009, mortality trends were relatively stable or declining in most groups. Black or African American individuals showed a significant decline with an APC of –1.67% (95% CI: –2.88 to –0.44, *p* = 0.0261), and White individuals experienced a modest yet significant decrease of –1.09% (95% CI: –1.79 to –0.39, *p* = 0.0057). Asian or Pacific Islander individuals also showed a declining trend (APC: –2.77%, 95% CI: –5.58 to +0.14), though this was not statistically significant (*p* = 0.0944). In contrast, American Indian or Alaska Native and Hispanic or Latino individuals experienced nonsignificant changes, with APCs of +0.52% (95% CI: –6.79 to +8.41, *p* = 0.9141) and –0.53% (95% CI: –2.16 to +1.12, *p* = 0.5099), respectively. The decade from 2010 to 2020 saw a concerning reversal in these trends, with all five groups experiencing statistically significant increases in mortality, except for American Indian or Alaska Native individuals, whose increase did not reach significance. White individuals showed the most pronounced rise, with an APC of +5.09% (95% CI: +4.18 to +6.00, *p* < 0.0001), followed by Black or African American individuals, who saw a sharp increase of +3.88% (95% CI: +2.94 to +4.84, *p* < 0.0001). Similarly, Hispanic or Latino individuals experienced a significant rise in AAMRs with an APC of +4.29% (95% CI: +2.94 to +5.66, *p* < 0.0001), and Asian or Pacific Islander individuals had an APC of +4.62% (95% CI: +1.60 to +7.74, *p* = 0.0145). The increase among American Indian or Alaska Native individuals, while notable at +2.89%, did not reach statistical significance (95% CI: –0.40 to +6.27, *p* = 0.1237) (Table 2; Figure 2).

### 3.4 Trends by Metropolitan (Urbanization) Status

From 1999 to 2020, hyperkalemia‐related SCD rates were consistently higher in nonmetropolitan areas compared to metropolitan regions. In metropolitan areas, the AAMR ranged from 0.59 (95% CI: 0.54–0.64) per 100 000 in 2008 to 1.14 (95% CI: 1.08–1.20) in 2020. Meanwhile, nonmetropolitan regions exhibited a broader and consistently higher range, with AAMRs rising from 0.60 (95% CI: 0.55–0.66) in 2005 to 1.40 (95% CI: 1.32–1.49) in 2020 (Figure 3).

**Figure 3:**
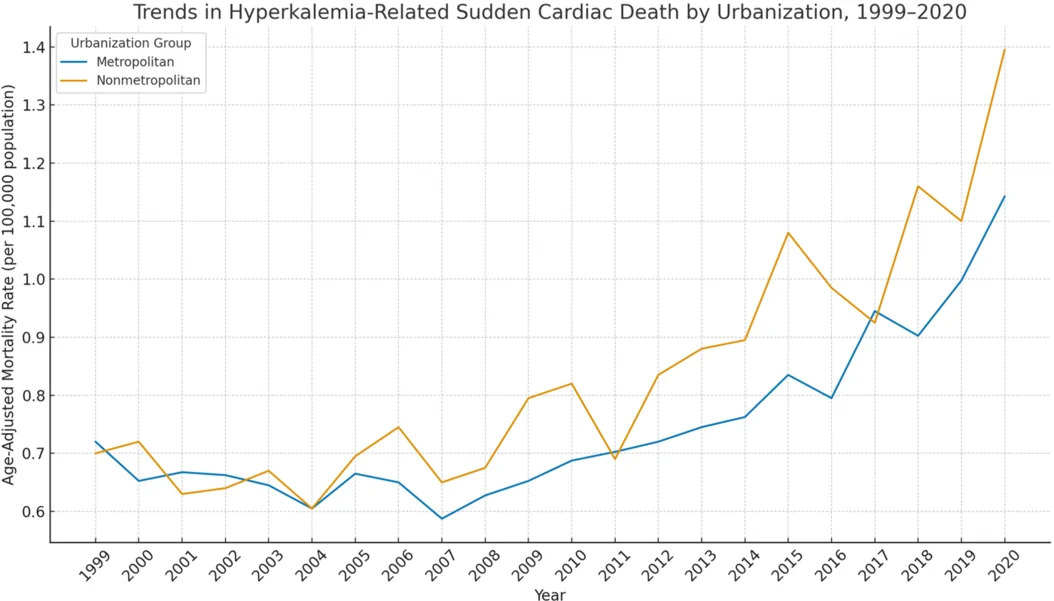
Trends in age‐adjusted mortality rates for hyperkalemia‐related sudden cardiac death stratified by metropolitan and nonmetropolitan residence among older adults, 1999–2020.

Trend segmentation revealed distinct phases. Between 1999 and 2009, metropolitan regions experienced a borderline significant decline in mortality, with an APC of –0.94% (95% CI: –1.78% to –0.09%, *p* = 0.0584). In contrast, nonmetropolitan areas showed a nonsignificant upward trend with an APC of +0.74% (95% CI: –0.74% to +2.25%, *p* = 0.3546). In the decade from 2010 to 2020, both groups demonstrated statistically significant increases. The APC for metropolitan areas was +4.78% (95% CI: +3.77% to +5.80%, *p* < 0.0001), while nonmetropolitan areas saw a slightly steeper annual increase of +5.32% (95% CI: +3.48% to +7.19%, *p* = 0.0003). These findings emphasize a nationwide upward trajectory in hyperkalemia‐related SCD, with a disproportionate impact on rural populations (Table 2; Figure 3).

### 3.5 Trends by Census Region

Between 1999 and 2020, regional disparities were observed in the AAMRs for hyperkalemia‐related SCD across the four US Census regions. In the Midwest, mortality rates were consistently higher than in other regions. AAMRs rose from 0.93 (95% CI: 0.85–1.01) per 100 000 in 1999 to 1.81 (95% CI: 1.72–1.91) in 2020. The South followed closely, increasing from 0.87 (95% CI: 0.82–0.92) to 1.55 (95% CI: 1.49–1.61) over the same period. The West showed a unique pattern, beginning with a relatively high rate of 0.92 (95% CI: 0.85–0.99) in 1999, followed by a dip in the early 2000s, then a rise to 1.46 (95% CI: 1.38–1.54) by 2020. Meanwhile, the Northeast consistently reported the lowest AAMRs, starting at 0.78 (95% CI: 0.69–0.87) in 1999 and reaching 1.22 (95% CI: 1.14–1.30) in 2020. Despite these lower absolute values, a clear upward trend was still evident (Figure 4).

**Figure 4:**
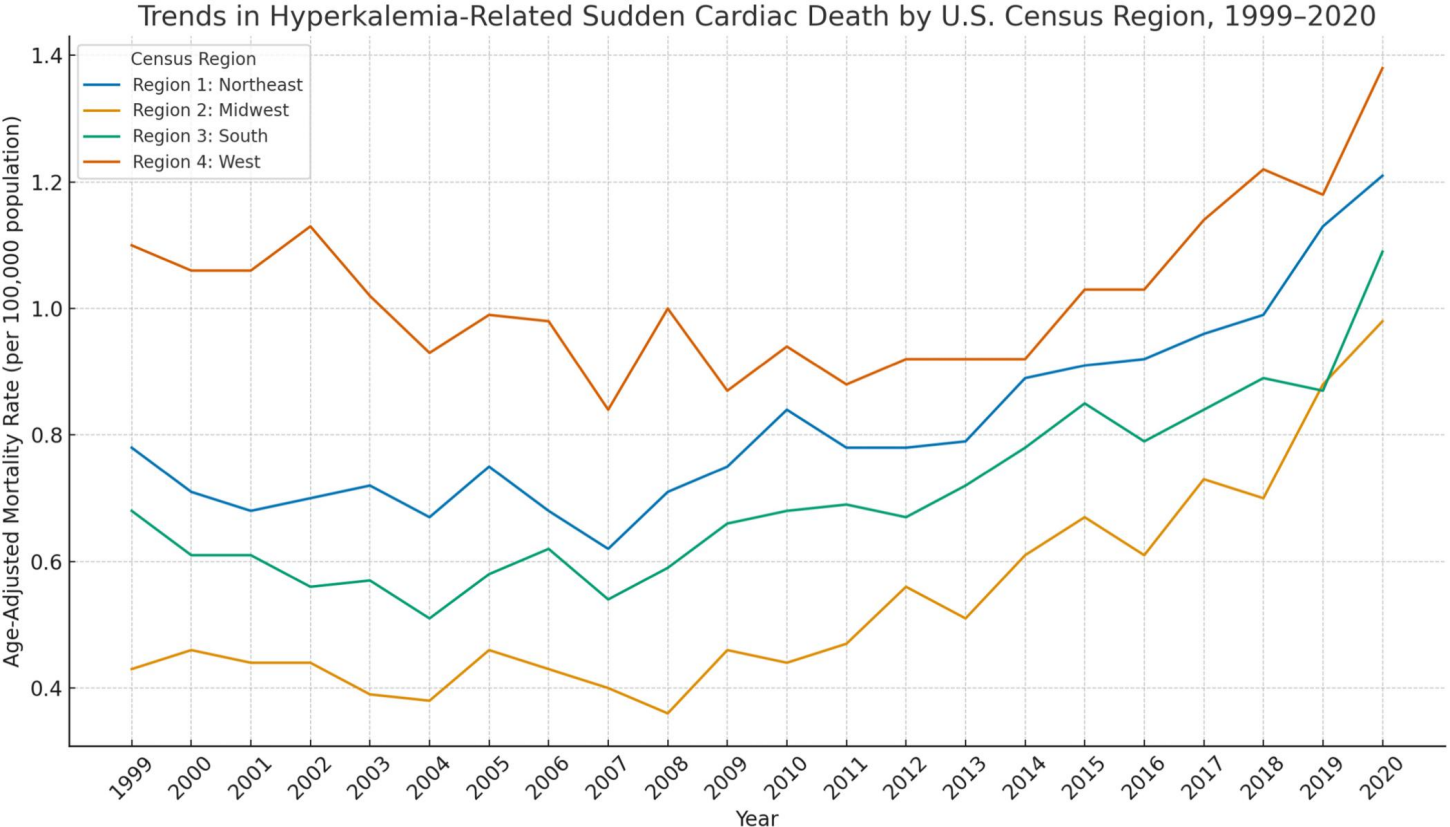
Trends in age‐adjusted mortality rates for hyperkalemia‐related sudden cardiac death stratified by US census region among older adults, 1999–2020.

Trend analysis revealed two distinct time segments: 1999–2009 and 2010–2020. From 1999 to 2009, all regions either declined or remained stable. The West was the only region with a statistically significant decrease, with an APC of –2.17% (95% CI: –3.28 to –1.06, *p* = 0.0043). The Northeast, Midwest, and South exhibited nonsignificant changes with APCs of –0.44% (95% CI: –1.65 to +0.77, *p* = 0.4900), –0.73% (95% CI: –2.31 to +0.87, *p* = 0.3900), and –0.39% (95% CI: –2.00 to +1.25, *p* = 0.6526), respectively. From 2010 to 2020, all regions showed statistically significant increases. The Midwest experienced the most rapid rise, with an APC of +7.45% (95% CI: +5.94 to +8.97, *p* < 0.0001). The West followed with an APC of +4.16% (95% CI: +3.03 to +5.30, *p* < 0.0001), while the Northeast rose at +4.12% (95% CI: +2.97 to +5.29, *p* = 0.0001) (Table 2; Figure 4).

### 3.6 Trends by State

#### 3.6.1 State‐Level Variation in Hyperkalemia‐Related SCD (1999–2020)

Across the United States, AAMRs for hyperkalemia‐related SCD varied markedly between states over the study period. The highest AAMR was observed in Nevada, with a rate of 1.58 per 100 000 population (95% CI: 1.45–1.71), followed by Rhode Island at 1.39 (95% CI: 1.22–1.57), and California at 1.22 (95% CI: 1.19–1.25). These elevated rates may reflect a combination of high comorbidity burden, regional practice patterns, or differences in healthcare access and coding practices (Figure 5).

**Figure 5:**
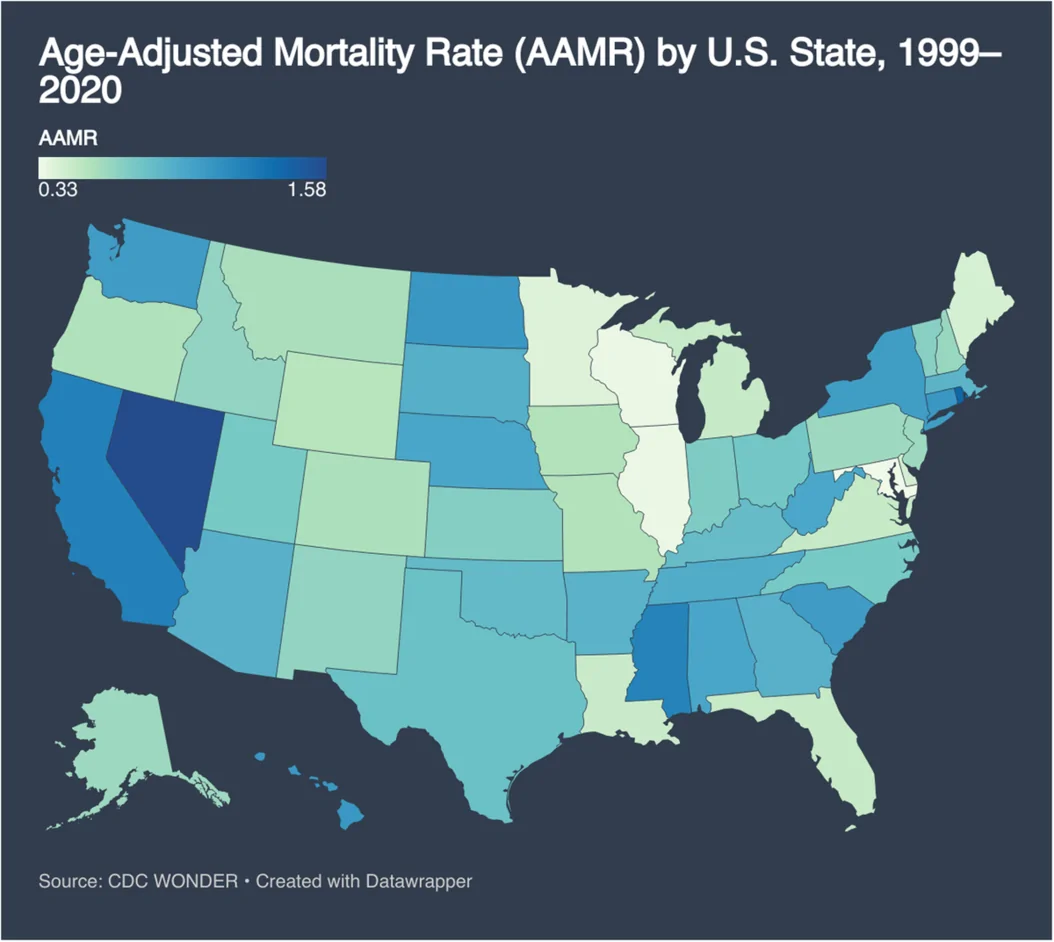
Geographic distribution of age‐adjusted mortality rates for hyperkalemia‐related sudden cardiac death by US state among older adults, 1999–2020.

In contrast, the lowest AAMRs were recorded in Illinois, with a rate of 0.35 per 100 000 (95% CI: 0.32–0.38), and Wisconsin, also at 0.35 (95% CI: 0.31–0.38). Other states with notably low mortality rates included Maryland at 0.33 (95% CI: 0.30–0.37) and Virginia at 0.48 (95% CI: 0.44–0.52). These differences likely reflect variation in chronic disease management, healthcare infrastructure, and preventive care engagement. The more than fourfold difference between the highest and lowest AAMRs highlights significant geographic disparities in outcomes related to hyperkalemia‐associated cardiac events (Figure 5).

### 3.7 Trends by 10‐Year Age Group

CRs for hyperkalemia‐related SCD rose with advancing age and demonstrated distinct temporal patterns across age groups. In 1999, the CR among individuals aged 25–34 years was 0.09 per 100 000 (95% CI: 0.07–0.11), while it increased steadily in older groups, reaching 1.64 (95% CI: 1.59–1.70) in the 65–74 age group and 5.73 (95% CI: 5.61–5.85) among those aged 75–84 years. The highest CR was observed in the ≥ 85 years group, at 13.46 (95% CI: 13.23–13.69). This age‐dependent gradient persisted in 2020, with CRs rising to 0.20 (95% CI: 0.17–0.23) in the 25–34 group, 3.06 (95% CI: 2.97–3.15) in the 65–74 group, and 11.80 (95% CI: 11.63–11.97) in the ≥ 85 group. These findings highlight the disproportionate burden of hyperkalemia‐related SCD among older adults, though modest increases in younger age groups were also evident (Figure 6).

**Figure 6:**
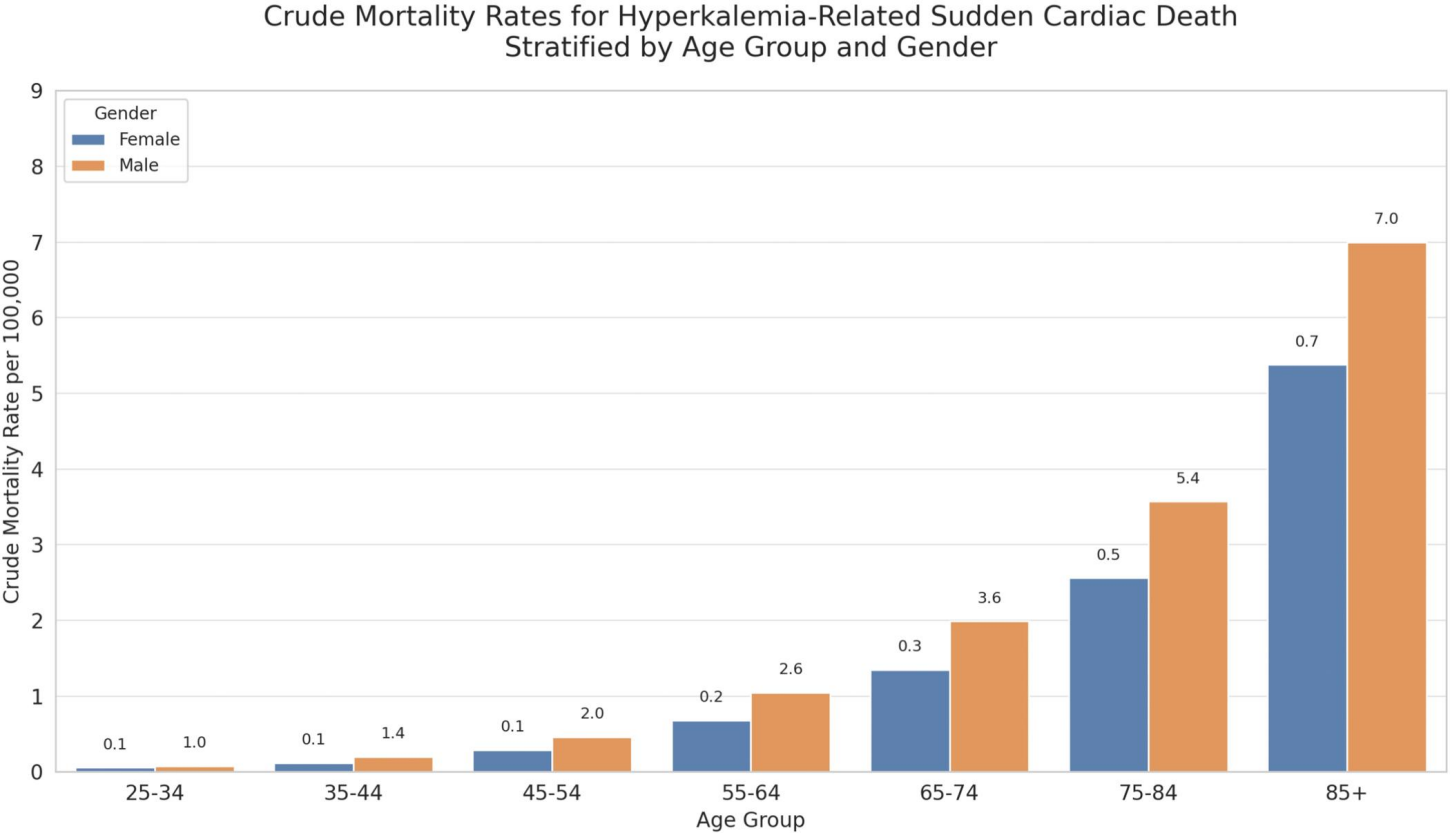
Trends in crude mortality for hyperkalemia‐related sudden cardiac death stratified by 10‐year age group and gender among older adults, 1999–2020.

The segmented AAPC analysis revealed a shift in trends over time. From 1999 to 2009, most age groups experienced stable or declining mortality. For example, the 25–34 age group had a nonsignificant decline with an APC of –0.81% (95% CI: –3.25 to +1.68, *p* = 0.5433), while the 35–44 group declined at –1.16% (95% CI: –3.38 to +1.11, *p* = 0.3400). The 45–54 group was relatively stable with an APC of +1.32% (95% CI: –0.18 to +2.84, *p* = 0.1187). However, from 2010 to 2020, a significant upward trend emerged in multiple age groups. The 25–34 age group exhibited a statistically significant APC of +6.84% (95% CI: +2.91 to +10.91, *p* = 0.0071), and the 35–44 group showed an even greater increase of +8.75% (95% CI: +6.44 to +11.12, *p* < 0.0001). These findings point to a concerning rise in hyperkalemia‐related SCD among younger adults in the most recent decade (Table 2; Figure 6).

## 4 Discussion

In this national, population‐based study spanning over two decades, we observed a striking increase in the mortality burden of hyperkalemia‐related SCD among US adults aged ≥ 25 years. Between 1999 and 2020, 36 607 deaths were attributed to this cause, and the AAMR more than doubled, rising from 0.65 to 1.32 per 100 000. This trajectory represents a significant public health concern and may reflect both a growing clinical burden and heightened recognition of hyperkalemia's arrhythmogenic potential.

Temporal analysis revealed a nonlinear trend. From 1999 to 2009, mortality remained relatively stable, with a borderline‐significant APC, suggesting some consistency in outcomes. However, beginning in 2010, mortality rates rose sharply, with an APC of +5.02% per year, marking a notable epidemiologic shift. This inflection may coincide with an aging population, increasing prevalence of chronic disease, and broader use of medications that elevate serum potassium, particularly RAAS inhibitors [6, 15].

This overall trend masked significant disparities by race, sex, geography, and urbanization. Black individuals exhibited the highest AAMRs, increasing from 1.24 to 2.30 per 100 000, compared to an increase from 0.69 to 1.31 among White individuals. This disparity likely reflects a higher burden of comorbidities—such as CKD, hypertension, and diabetes—as well as unequal access to high‐quality care and systemic inequities [16, 17]. Emerging evidence also points to possible genetic differences in renal potassium handling and RAAS activity that may contribute to increased vulnerability in this population [18]. Given the strong clinical overlap between hyperkalemia, CKD, HF, and DM, disentangling their individual contributions to SCD is inherently challenging. Death certificate data lack granularity on disease severity, treatment status, or temporal sequencing of events. However, the repeated documentation of hyperkalemia as a contributing cause of death across thousands of cases suggests that certifying physicians recognized it as a clinically meaningful factor. Rather than acting in isolation, hyperkalemia likely amplifies the arrhythmogenic potential of existing comorbidities through synergistic physiological stress. Males consistently had higher mortality rates than females, with a steeper post‐2010 rise, aligning with broader cardiovascular mortality patterns and a higher incidence of ventricular arrhythmias in men [19, 20]. Geographic differences were also pronounced. The Midwest and South exhibited higher baseline rates and steeper increases over time, consistent with regional disparities in chronic disease burden and healthcare access [21, 22]. Individuals in nonmetropolitan areas showed consistently higher AAMRs compared to those in metropolitan regions, likely due to challenges in healthcare delivery, emergency response, and chronic disease management in rural settings [23]. While mortality rates rose sharply with age, younger adults (25–44 years) also experienced significant increases in APC since 2010, suggesting the need for heightened clinical awareness in lower‐risk age groups.

At the cellular level, hyperkalemia disrupts cardiac electrophysiology by lowering the resting membrane potential, which shortens action potential duration and increases the risk of fatal arrhythmias such as ventricular tachycardia or fibrillation. Sudden elevations in serum potassium can result in conduction blocks, bradyarrhythmias, and ultimately asystole or pulseless electrical activity [24, 25]. Clinically, this deterioration is marked by a recognizable ECG progression—peaked T waves, PR prolongation, QRS widening, sine‐wave morphology—culminating in cardiac arrest if untreated [2]. These effects are particularly dangerous in patients with underlying cardiovascular disease, whose arrhythmic thresholds may be lower.

The sharp rise in mortality after 2010 likely reflects multiple converging trends. The aging US population has brought with it a growing prevalence of CKD, HF, and DM—three major risk factors for impaired renal potassium excretion [26]. Meanwhile, RAAS inhibitors have become standard therapy in managing these conditions. While effective in slowing disease progression and improving outcomes, these agents impair renal potassium handling and can precipitate hyperkalemia, particularly in patients with reduced glomerular filtration [6]. In clinical practice, inadequate monitoring after initiating or adjusting these therapies can lead to preventable complications [8]. Healthcare access barriers further compound the issue: patients in rural or underserved areas may have limited access to laboratory testing, specialist consultation, and early interventions for hyperkalemia [23]. Additionally, growing trends in dietary supplementation and “heart‐healthy” diets may increase potassium intake without adequate consideration of individual kidney function, potentially heightening subclinical hyperkalemia risk in vulnerable individuals.

These findings have important implications for clinical practice and public health strategy. Hyperkalemia is a reversible condition, yet if unrecognized or mismanaged, it can rapidly escalate to fatal arrhythmia. Proactive risk stratification is essential, particularly for individuals with impaired renal function or those receiving RAAS inhibitors. Although these agents provide substantial cardiovascular and renal benefit, their use necessitates regular laboratory surveillance—especially in older adults or those with borderline renal function [6]. However, evidence indicates that potassium monitoring in real‐world settings is often delayed or inconsistent, leaving patients exposed to unnecessary risk [8].

Newer potassium‐binding therapies—such as patiromer and sodium zirconium cyclosilicate—offer safer, more tolerable options for managing chronic hyperkalemia. Their expanded use could allow patients to remain on life‐prolonging RAAS therapies without compromising safety [27]. Yet, cost, access, and provider familiarity continue to limit their broader adoption. Addressing these gaps will require increased clinician education and policy initiatives to improve patient access. On a systemic level, persistent disparities in mortality—particularly in rural and underserved populations—highlight the need for equitable healthcare delivery models. Integrating telehealth, mobile labs, and community health workers into chronic disease management frameworks may help bridge the gap. Public education efforts targeting older adults and high‐risk populations on hyperkalemia triggers—such as dietary sources and medication effects—could further reduce delays in care. Finally, embedding potassium monitoring into healthcare quality metrics (e.g., CMS Star Ratings) may incentivize better surveillance and earlier intervention [28].

The observed increase in hyperkalemia‐related SCD among adults aged 25–44 years is particularly concerning and suggests the emergence of underrecognized risk factors in this demographic. While traditionally considered a complication of aging or chronic disease, recent data suggest that younger individuals may be increasingly vulnerable due to evolving patterns in drug use, healthcare access, and clinical practice. Recreational substances such as 3,4‐methylenedioxymethamphetamine (MDMA or “ecstasy”) and synthetic cannabinoids have been associated with severe hyperkalemia and malignant arrhythmias, even in individuals without prior cardiovascular disease [29, 30]. In parallel, iatrogenic causes—including inappropriate potassium supplementation, insufficient renal function monitoring, and prescribing errors—have been identified as triggers for fatal hyperkalemia in younger outpatient populations [21]. Additionally, the rising use of over‐the‐counter dietary supplements, “clean eating” trends promoting potassium‐rich foods, and unsupervised herbal remedies may also contribute to subclinical potassium accumulation, especially in undiagnosed CKD cases [31]. Social determinants such as delayed care‐seeking, poor access to primary care, and diagnostic overshadowing in younger adults may further exacerbate the risk.

Taken together, our findings emphasize that while hyperkalemia is a physiologically simple abnormality, its mortality impact is profound when overlooked. Effective prevention will require coordinated action across multiple levels: clinical decision‐making, patient education, public health infrastructure, and policy reform.

## 5 Limitations

This study acknowledges several important limitations. First, the use of death certificate data introduces the potential for underreporting or misclassification of hyperkalemia and SCD, particularly across demographic groups where diagnostic accuracy may vary [32, 33]. The reliance on ICD‐10 codes, while standardized, limits the clinical context available and precludes access to key variables such as comorbidities, laboratory‐confirmed serum potassium levels, medication use (e.g., RAAS inhibitors), and severity of underlying conditions—all of which may significantly influence both the risk and timing of hyperkalemia‐related mortality. Importantly, causality cannot be firmly established from death certificate data. While we identified deaths where hyperkalemia was listed as a contributing cause to SCD, this classification is subject to misclassification bias, as the role of hyperkalemia in triggering the terminal event may be inferred rather than confirmed. The presence of multiple comorbidities, such as CKD or HF, further complicates causal attribution.

Furthermore, the absence of data on care processes—such as the timing of medical intervention, location of death (in‐hospital vs. out‐of‐hospital), or emergency response times—prevents a comprehensive understanding of how delays in recognition or treatment may contribute to observed mortality patterns. The retrospective, observational nature of this study also limits control over data collection practices and increases susceptibility to unmeasured confounding factors.

For example, we were unable to account for the introduction of newer potassium‐lowering agents or regional variations in practice patterns over time. However, we cannot entirely exclude the possibility that improved recognition or recording of hyperkalemia in later years may have contributed in part to the observed increase in mortality.

Recognizing these limitations is essential for interpreting our findings within their proper context. Future research should seek to incorporate more detailed clinical and treatment data—such as medication regimens, potassium trajectories, and health system performance indicators—to better clarify causal pathways. Integrating qualitative methods or electronic health record–linked data may help illuminate patient‐level, provider‐level, and system‐level factors driving disparities. In addition, prospective or longitudinal cohort studies would offer a more robust framework to assess the temporal dynamics and modifiable contributors to hyperkalemia‐related SCD. By refining methodology and addressing these data gaps, future investigations can build upon the present findings to guide more effective, equitable, and targeted interventions.

## 6 Conclusion

In this nationwide analysis spanning more than two decades, we identified a substantial and accelerating increase in hyperkalemia‐related SCD among older adults in the United States. The AAMRs more than doubled between 1999 and 2020, with a sharp inflection beginning around 2010. These trends were not evenly distributed: males, Black individuals, residents of the South and Midwest, and those in nonmetropolitan areas experienced disproportionately higher mortality burdens and steeper annual increases. While older adults carried the highest absolute risk, our findings also revealed rising trends in younger age groups, signaling a widening public health concern.

This evolving epidemiologic profile of hyperkalemia‐related SCD highlights the urgent need for targeted clinical strategies and health system interventions. Prioritizing potassium monitoring, improving access to chronic disease management, and ensuring equitable care delivery are essential to reversing these trends. As hyperkalemia is a modifiable risk factor, it represents a clear and actionable target in the prevention of SCD. Efforts should focus on risk prediction, timely therapeutic intervention, and eliminating the structural barriers that continue to place vulnerable populations at heightened risk.

## Author Contributions

**Ibrahim Nagmeldin Hassan:** conceptualization, methodology, project administration, visualization, writing – original draft, writing – review and editing. **Mohamed Ibrahim:** conceptualization, formal analysis, visualization, writing – original draft, writing – review and editing. **Siddig Yaqub:** project administration, validation, writing – original draft, writing – review and editing. **Muhsin Ibrahim:** writing – original draft, writing – review and editing. **Nagmeldin Abuassa:** writing – original draft, writing – review and editing. All authors read and approved the final manuscript.

## Funding

The authors have nothing to report.

## Ethics Statement

The authors have nothing to report.

## Consent

The authors have nothing to report.

## Conflicts of Interest

The authors declare no conflicts of interest.

## Supporting information


**Table S1:** ICD‐10 Codes Used to Identify Hyperkalemia‐Related Sudden Cardiac Death During the Study Period (1999–2020).
**Table S2:** Strengthening the Reporting of Observational Studies in Epidemiology (STROBE) Checklist.

## Data Availability

The data that support the findings of this study are openly available in CDC WONDER at https://wonder.cdc.gov/, reference number N/A.

## References

[1] B. F. Palmer and D. J. Clegg , “Physiology and Pathophysiology of Potassium Homeostasis,” Advances in Physiology Education 40, no. 4 (December 2016): 480–490, 10.1152/advan.00121.2016. 27756725

[2] C. G. Acker , J. P. Johnson , P. M. Palevsky , and A. Greenberg , “Hyperkalemia in Hospitalized Patients: Causes, Adequacy of Treatment, and Results of an Attempt to Improve Physician Compliance With Published Therapy Guidelines,” Archives of Internal Medicine 158, no. 8 (April 1998): 917–924, 10.1001/archinte.158.8.917. 9570179

[3] M. R. Weir and M. Rolfe , “Potassium Homeostasis and Renin‐Angiotensin‐Aldosterone System Inhibitors,” Clinical Journal of the American Society of Nephrology 5, no. 3 (March 2010): 531–548, 10.2215/CJN.07821109. 20150448

[4] A. Mattu , W. J. Brady , and D. A. Robinson , “Electrocardiographic Manifestations of Hyperkalemia,” American Journal of Emergency Medicine 18, no. 6 (October 2000): 721–729, 10.1053/ajem.2000.7344. 11043630

[5] J. P. Fauvel , F. Gueyffier , L. Thijs , and M. Ducher , “Combined Effect of Renal Function and Serum Potassium Level in Sudden Cardiac Death in Aging Hypertensive Subjects,” Hypertension Research 41, no. 6 (June 2018): 469–474, 10.1038/s41440-018-0035-3. 29632405

[6] L. M. Einhorn , M. Zhan , V. D. Hsu , et al., “The Frequency of Hyperkalemia and Its Significance in Chronic Kidney Disease,” Archives of Internal Medicine 169, no. 12 (June 2009): 1156–1162, 10.1001/archinternmed.2009.132. PMC3544306 19546417

[7] J. F. Neuenschwander , A. R. Silverstein , C. L. Teigland , et al., “The Increased Clinical and Economic Burden of Hyperkalemia in Medicare Patients Admitted to Long‐Term Care Settings,” Advances in Therapy 40, no. 3 (March 2023): 1204–1223, 10.1007/s12325-022-02420-x. PMC9988794 36652174

[8] J. Luo , S. M. Brunelli , D. E. Jensen , and A. Yang , “Association Between Serum Potassium and Outcomes in Patients With Reduced Kidney Function,” Clinical Journal of the American Society of Nephrology 11, no. 1 (January 2016): 90–100, 10.2215/CJN.01730215. PMC4702219 26500246

[9] “Multiple Cause of Death 1999–2019 on CDC WONDER Online Database, Released in 2020. Data Are From the Multiple Cause of Death Files, 1999‐2019, as Compiled From Data Provided by the 57 Vital Statistics Jurisdictions Through the Vital Statistics Cooperative Program,” Centers for Disease Control and Prevention, National Center for Health Statistics, accessed March 24, 2025, http://wonder.cdc.gov/mcd-icd10.html.

[10] “Free 2019 ICD‐10‐CM Codes,” Icd10data.com, Published 2019, https://www.icd10data.com/ICD10CM/Codes/Changes/.

[11] E. von Elm , D. G. Altman , M. Egger , S. J. Pocock , P. C. Gøtzsche , and J. P. Vandenbroucke , “The Strengthening the Reporting of Observational Studies in Epidemiology (STROBE) Statement: Guidelines for Reporting Observational Studies,” Journal of Clinical Epidemiology 61, no. 4 (April 2008): 344–349, 10.1016/j.jclinepi.2007.11.008. 18313558

[12] D. D. Ingram and S. J. Franco , “2013 NCHS Urban‐Rural Classification Scheme for Counties,” Vital and Health Statistics. Series 2, Data Evaluation and Methods Research 2 (2014): 1–73. 24776070

[13] “Joinpoint Regression Program, Version 5.0.2,” Statistical Methodology and Applications Branch, Surveillance Research Program, National Cancer Institute, May 2023.

[14] H. J. Kim , M. P. Fay , E. J. Feuer , and D. N. Midthune , “Permutation Tests for Joinpoint Regression With Applications to Cancer Rates,” Statistics in Medicine 19, no. 3 (February 2000): 335–351, 10.1002/(sici)1097-0258(20000215)19:3<335::aid-sim336>3.0.co;2-z. 10649300

[15] C. P. Kovesdy , “Management of Hyperkalaemia in Chronic Kidney Disease,” Nature Reviews Nephrology 10, no. 11 (November 2014): 653–662, 10.1038/nrneph.2014.168. 25223988

[16] P. Medicine , “Racial Disparities in Sudden Cardiac Death Rates Cannot Be Explained by Known Risk Factors,” 2018, https://www.pennmedicine.org/news/news-releases/2018/november/racial-disparities-in-sudden-cardiac-death-rates-cannot-be-explained-by-known-risk-factors.

[17] S. B. Nicholas , K. Kalantar‐Zadeh , and K. C. Norris , “Racial Disparities in Kidney Disease Outcomes,” Seminars in Nephrology 33, no. 5 (September 2013): 409–415, 10.1016/j.semnephrol.2013.07.002. PMC3983362 24119846

[18] C. M. Clase , J. J. Carrero , D. H. Ellison , et al., “Potassium Homeostasis and Management of Dyskalemia in Kidney Diseases: Conclusions From a Kidney Disease: Improving Global Outcomes (KDIGO) Controversies Conference,” Kidney International 97, no. 1 (January 2020): 42–61, 10.1016/j.kint.2019.09.018. 31706619

[19] S. S. Chugh , J. Jui , K. Gunson , et al., “Current Burden of Sudden Cardiac Death: Multiple Source Surveillance Versus Retrospective Death Certificate‐Based Review in a Large US Community,” Journal of the American College of Cardiology 44, no. 6 (September 2004): 1268–1275, 10.1016/j.jacc.2004.06.029. 15364331

[20] V. Regitz‐Zagrosek and C. Gebhard , “Gender Medicine: Effects of Sex and Gender on Cardiovascular Disease Manifestation and Outcomes,” Nature Reviews Cardiology 20, no. 4 (April 2023): 236–247, 10.1038/s41569-022-00797-4. PMC9628527 36316574

[21] S. Sidney , C. P. Quesenberry , M. G. Jaffe , et al., “Recent Trends in Cardiovascular Mortality in the United States and Public Health Goals,” JAMA Cardiology 1, no. 5 (August 2016): 594–599, 10.1001/jamacardio.2016.1326. 27438477

[22] G. A. Mensah , G. S. Wei , P. D. Sorlie , et al., “Decline in Cardiovascular Mortality: Possible Causes and Implications,” Circulation Research 120, no. 2 (January 2017): 366–380, 10.1161/CIRCRESAHA.116.309115. PMC5268076 28104770

[23] E. Moy , M. C. Garcia , B. Bastian , et al., “Leading Causes of Death in Nonmetropolitan and Metropolitan Areas‐ United States, 1999‐2014,” MMWR. Surveillance Summaries 66, no. 1 (January 2017): 1–8, 10.15585/mmwr.ss6601a1. PMC5829895 28081058

[24] D. B. Mount and K. Zandi‐Nejad , “Disorders of Potassium Balance,” in Brenner & Rector's the Kidney, 10th ed. (Elsevier, 2016).

[25] J. N. Weiss , Z. Qu , and K. Shivkumar , “Electrophysiology of Hypo‐ and Hyperkalemia,” Circulation: Arrhythmia and Electrophysiology 10, no. 3 (2017): e004667. 10.1161/CIRCEP.116.004667 PMC5399982 28314851

[26] “USRDS Annual Data Report: Epidemiology of Kidney Disease in the United States,” United States Renal Data System, 2022. 10.1053/j.ajkd.2022.12.001 PMC10807034 36822739

[27] G. L. Bakris , B. Pitt , M. R. Weir , et al., “Effect of Patiromer on Serum Potassium Level in Patients With Hyperkalemia and Diabetic Kidney Disease: The AMETHYST‐DN Randomized Clinical Trial,” Journal of the American Medical Association 314, no. 2 (July 2015): 151–161, 10.1001/jama.2015.7446. 26172895

[28] “Medicare Star Ratings Technical Notes,” Centers for Medicare & Medicaid Services (CMS), https://www.cms.gov.

[29] J. A. Henry , K. J. Jeffreys , and S. Dawling , “Toxicity and Deaths From 3,4‐Methylenedioxymethamphetamine (‘Ecstasy’),” Lancet 340, no. 8816 (August 1992): 384–387, 10.1016/0140-6736(92)91469-o. 1353554

[30] Z. D. Cooper , “Adverse Effects of Synthetic Cannabinoids: Management of Acute Toxicity and Withdrawal,” Current Psychiatry Reports 18, no. 5 (May 2016): 52, 10.1007/s11920-016-0694-1. PMC4923337 27074934

[31] A. C. Brown , “Kidney Toxicity Related to Herbs and Dietary Supplements: Online Table of Case Reports. Part 3 of 5 Series,” Food and Chemical Toxicology 107, no. pt. A (September 2017): 502–519, 10.1016/j.fct.2016.07.024. 28755953

[32] K. A. Myers and D. R. Farquhar , “Improving the Accuracy of Death Certification,” CMAJ: Canadian Medical Association Journal = Journal de l'Association Medicale Canadienne 158, no. 10 (May 1998): 1317–1323. PMC1229326 9614825

[33] S. S. Chugh , K. Reinier , C. Teodorescu , et al., “Epidemiology of Sudden Cardiac Death: Clinical and Research Implications,” Progress in Cardiovascular Diseases 51, no. 3 (November/December 2008): 213–228, 10.1016/j.pcad.2008.06.003. PMC2621010 19026856

