# Supplementary material for: Trends in Hyperkalemia‐Related Sudden Cardiac Death Among Older Adults in the United States, 1999–2020
Source: Clin Cardiol. 2026 Oct 6;49(10):e70483. doi: 10.1002/clc.70483 (PMC13640976; doi:10.1002/clc.70483)
Supplement: Supplementary file 1 [file CLC-49-e70483-s001.docx]

**Table S1. ICD-10 Codes Used to Identify Hyperkalemia-Related Sudden Cardiac Death During the Study Period (1999–2020)**

| **ICD-10 codes** | **Definition** |
| --- | --- |
| E87.5 | Hyperkalemia |
| I46.1 | Sudden cardiac death, so described |
| I46.9 | Sudden cardiac death, unspecified |

**Table S2. Strengthening the Reporting of Observational Studies in Epidemiology (STROBE) Checklist**

|  | **Item No** | **Recommendation** | **Section or**  **page number** |
| --- | --- | --- | --- |
| **Title and abstract** | 1 | (*a*) Indicate the study’s design with a commonly used term in  the title or the abstract | 1 |
|  |  | (*b*) Provide in the abstract an informative and balanced | 2 |
|  |  | summary of what was done and what was found |  |
| **Introduction** |  |  |  |
| Background/rationale | 2 | Explain the scientific background and rationale for the | 3 |
|  |  | investigation being reported |  |
| Objectives | 3 | State specific objectives, including any prespecified | 3 |
|  |  | hypotheses |  |
| **Methods** |  |  |  |
| Study design | 4 | Present key elements of study design early in the paper | 4 |
| Setting | 5 | Describe the setting, locations, and relevant dates, including | 4 |
|  |  | periods of recruitment, exposure, follow-up, and data |  |
|  |  | collection |  |
| Participants | 6 | (*a*) Give the eligibility criteria, and the sources and methods | 4 |
|  |  | of selection of participants |  |
| Variables | 7 | Clearly define all outcomes, exposures, predictors, potential | 4 |
|  |  | confounders, and effect modifiers. Give diagnostic criteria, if |  |
|  |  | applicable |  |
| Data sources/ | 8* | For each variable of interest, give sources of data and details | 4 |
| measurement |  | of methods of assessment (measurement). Describe |  |
|  |  | comparability of assessment methods if there is more than |  |
|  |  | one group |  |
| Bias | 9 | Describe any efforts to address potential sources of bias | 4 |
| Study size | 10 | Explain how the study size was arrived at | 4 |
| Quantitative variables | 11 | Explain how quantitative variables were handled in the | 4 |
|  |  | analyses. If applicable, describe which groupings were |  |
|  |  | chosen and why |  |
| Statistical methods | 12 | (*a*) Describe all statistical methods, including those used to | 5 |
|  |  | control for confounding |  |
|  |  | (*b*) Describe any methods used to examine subgroups and | 5 |
|  |  | interactions |  |
|  |  | (c) Explain how missing data were addressed | 5 |
|  |  | (*d*) If applicable, describe analytical methods taking account | Not applicable |
|  |  | of sampling strategy |  |
|  |  | (*e*) Describe any sensitivity analyses | Not applicable |
| **Results** |  |  |  |
| Participants | 13* | (a) Report numbers of individuals at each stage of study—eg | 5 |
|  |  | numbers potentially eligible, examined for eligibility, |  |
|  |  | confirmed eligible, included in the study, completing follow- |  |
|  |  | up, and analysed |  |
|  |  | (b) Give reasons for non-participation at each stage | 5 |
|  |  | (c) Consider use of a flow diagram | - |
| Descriptive data | 14* | (a) Give characteristics of study participants (eg | 5-8 |
|  |  | demographic, clinical, social) and information on exposures |  |
|  |  | and potential confounders |  |

|  |  | (b) Indicate number of participants with missing data for  each variable of interest | 5-8 |
| --- | --- | --- | --- |
| Outcome data | 15* | Report numbers of outcome events or summary measures | 5-8 |
| Main results | 16 | (*a*) Give unadjusted estimates and, if applicable, confounder- adjusted estimates and their precision (eg, 95% confidence  interval). Make clear which confounders were adjusted for and why they were included | 5-8 |
|  |  | (*b*) Report category boundaries when continuous variables were categorized | Not applicable |
|  |  | © If relevant, consider translating estimates of relative risk  into absolute risk for a meaningful time period | Not applicable |
| Other analyses | 17 | Report other analyses done—eg analyses of subgroups and  interactions, and sensitivity analyses | Not applicable |
| **Discussion** |  |  |  |
| Key results | 18 | Summarise key results with reference to study objectives | 9 |
| Limitations | 19 | Discuss limitations of the study, taking into account sources of potential bias or imprecision. Discuss both direction and  magnitude of any potential bias | 10-11 |
| Interpretation | 20 | Give a cautious overall interpretation of results considering objectives, limitations, multiplicity of analyses, results from  similar studies, and other relevant evidence | 10-11 |
| Generalisability | 21 | Discuss the generalisability (external validity) of the study  results | 10-11 |
| **Other information** |  |  |  |
| Funding | 22 | Give the source of funding and the role of the funders for the present study and, if applicable, for the original study on  which the present article is based | 12 |
